# Protein Supplementation with Low Fat Meat after Resistance Training: Effects on Body Composition and Strength

**DOI:** 10.3390/nu6083040

**Published:** 2014-08-04

**Authors:** Massimo Negro, Matteo Vandoni, Sara Ottobrini, Erwan Codrons, Luca Correale, Daniela Buonocore, Fulvio Marzatico

**Affiliations:** 1 Laboratory of Pharmacobiochemistry, Sports Nutrition and Nutriceuticals, University of Pavia, via A. Ferrata, 9-27100 Pavia, Italy; E-Mails: daniela.buonocore@unipv.it (D.B.); fulvio.marzatico@unipv.it (F.M.); 2 LAMA (Laboratory of Adapted Motor Activity)-CRIAMS, University of Pavia, via C. Forlanini, 2-27100 Pavia, Italy; E-Mails: matteo.vandoni@unipv.it (M.V.); sara.ottobrini01@ateneopv.it (S.O.); codrons.erwan@gmail.com (E.C.); luca.correale01@ateneopv.it (L.C.); 3 Department of Public Health, Experimental Medicine and Forensic Science, University of Pavia, via C. Forlanini, 2-27100 Pavia, Italy

**Keywords:** meat protein, resistance exercise, supplementation timing, lean body mass, muscle strength, muscle recovery

## Abstract

Beef is a nutrient-rich, high-quality protein containing all the essential amino acids in proportions similar to those found in human skeletal muscle. In order to investigate the efficacy of a beef supplementation strategy on strength and body composition, we recruited 26 young healthy adults to participate in a resistance-training program of eight weeks, based on the use of isotonic machines and free weights at 75% of one repetition maximum. Subjects were randomly divided into two groups, food group and control group, of 12 and 14 subjects respectively. Food group were supplemented after resistance training with a 135 g serving of lean beef (tinned meat), providing 20 g of protein and 1.7 g of fat. No supplementation was provided to control group. Fat mass, fat free mass, lean mass, assessed by bioelectrical impedance analyzer, and muscle strength, assessed by one repetition maximum test, were evaluated in all subjects both at the beginning (week 0) and at the end (week 8) of the study. Pre- and post-training differences were evaluated with paired *t*-tests while group differences for each outcome parameter was evaluated with independent *t*-tests. At the end of the study the food group showed a significantly decrease in fat mass (week 0: 15.0 ± 6.7 kg; week 8: 13.1 ± 7.6 kg; Δ: −1.9 ± 2.9 kg; *p* < 0.05) and a significantly increase in fat free mass (week 0: 52.8 kg ± 9.4; week 8: 55.1 kg ± 10.9; Δ: 2.3 ± 2.5 kg; *p* < 0.01). No significant differences in lean mass were found in either food group or control group. No significant differences in one repetition maximum tests were found between food group and control group. Tinned meat can be considered a nutrition strategy in addition to other proteins or amino acid supplements, but as with any other supplementation strategy, a proper nutrition plan must be coupled.

## 1. Introduction

In the last few decades, nutrition strategies based on protein and amino acid supplements have been used widely in resistance training protocols showing their effectiveness on muscle protein synthesis. Several findings [1] have shown that individuals who engage in resistance weight training, whether as professional athletes or to promote optimal physical outcomes, can benefit by these nutritional strategies in order to obtain different goals (maximize muscle hypertrophy and strength, preserve lean body mass during fat loss program, *etc.*). However, the timing (pre/post workout), type or amount of protein intake required to meet strength-training or body composition goals are not completely understood.

As recently discussed [2,3], protein timing is a popular dietary strategy designed to optimize the adaptive response to exercise. The strategy involves consuming protein around a training session (just before and/or immediately following) to facilitate muscular repair and remodeling, and thereby enhance post-exercise strength and hypertrophy-related adaptations [2,3]. A number of studies support the superiority of protein timing to stimulate an acute increase in muscle protein synthesis after resistance training [2,3], but literature has shown mixed results and a recent meta-analysis [4] does not support this theory.

Studies that match protein timing with muscle protein synthesis or muscle adaptation/hypertrophy mainly focus on trials that have used a variety of milk- and soy-based protein supplements as well as amino acids formulation [1,2,3,4]. Whey protein powders have received the most attention for their content of essential amino acids and leucine and therefore for their greater muscle anabolic value [1,2,3,4]. A typical post-workout dosage generally suggested for active adults and young athletes is about 20 g of whey protein, containing 8–10 g of essential amino acids [1,2,5]. It has been shown that ingestion of 5 g to 10 g of protein after resistance exercise is sufficient to stimulate higher rates of muscle protein synthesis than exercise alone; however, the response of muscle protein synthesis reached a plateau with the ingestion of 20 g of protein after exercise [6,7].

Other studies on protein timing have used whole-food protein sources (*i.e.*, cow and soy milk) coupled with resistance exercise [8,9], using amount of food containing about 20 g of protein (e.g., 500 mL milk). In general, results indicated that consumption of fat-free milk post-workout was statistically more effective than soymilk in promoting increases in muscle protein synthesis [8,9].

Very few studies have used meat (e.g., beef) to evaluate the effects of whole-food protein on the enhancement of muscle protein synthesis with a protein-timing model exercise related. Only two studies on this topic are available at present [10,11]. Beef is a nutrient-rich, high-quality protein containing all the essential amino acids in proportions similar to those found in human skeletal muscle [12]. Symson *et al.* [10] investigated the anabolic response of a bout of resistance exercise (6 sets of 8 repetitions of leg extension exercise at 80% of their one repetition maximum) in combination with a high quality protein-rich meal (340 g of beef, which contained 90 g protein) in older adults compared to their younger counterparts. The beef serving was administrated 60 min before the exercise, considering that meat is a slow-digested protein source. The combination of a protein rich meal and resistance exercise produces a robust acute synergistic effect on muscle protein synthesis, 2-fold greater than beef intake alone [13], with a similar response for young and older. More recently, Robinson *et al.* [11] demonstrate that a 170 g serving of lean beef, providing 36 g of protein, resulted in greater rates of muscle protein synthesis in middle-aged persons than smaller servings of 113 g and 57 g of beef (24 g and 12 g of protein, respectively) when administrated after 3 sets of knee extensions exercise (using a predetermined load to elicit failure within 8–10 repetitions). However, both studies described used a typical acute one-leg exercise protocol and it is still not known if “beef supplementation” can affect muscle adaptation during a classic weight-training program with different resistance exercises performed. Based on these data, the aim of the present study was to investigate the efficacy of a beef supplementation protocol on strength and body composition of young adults involved in a resistance-training program of eight weeks.

## 2. Experimental Section

### 2.1. Subjects

Forty healthy volunteers participated in the study. The subjects were recruited at the University of Pavia through advertisements posted on the main campus. Exclusion criteria included recent injuries, clinical conditions, medical treatments, and specific nutrition strategies or supplementation programs followed in the six months before the study, in order to change body composition or improve muscle performance. Inclusion criteria included a body mass index between 18.5 and 24.99 kg/m^2^, an age between 18 and 30 years old and no more than two training sessions per week as a program of habitual physical activity. Subjects were randomly divided into two groups, FG and CG, of 20 subjects each. However, 14 subjects were excluded during the study because they did not accurately respect the training design protocol. The characteristics of the subjects who completed the study (26 out of 40; 12 FG and 14 CG) are presented in Table 1. Written informed consent was obtained for all subjects. The study was approved by the Institutional Review Board at the University of Pavia.

### 2.2. Nutritional Assessment and Supplementation Protocol

Participants completed diet records prior to the start of the study to provide an estimate of habitual protein consumption. Protein intake of the participants was about 1.0 g/kg/day. For diet analysis we used a commercially available software program (MetaDieta^®^, ME.TE.DA s.r.l., San Benedetto Del Tronto, Italy). Volunteers were encouraged to maintain their normal diet during the entire time of study to avoid changing nutrition stimulus on muscle mass. FG were supplemented soon after every exercise session with a 135 g serving of lean beef (tinned meat), providing 20 g of protein and 1.7 g of fat; water was available to favor the swallowing. No supplementation was provided to CG.

### 2.3. Resistance Training Protocol

FG and CG followed a specific resistance training program designed by a certified strength and conditioning specialist. All subjects trained three times per week (Monday, Wednesday, and Friday), for a total of nine weeks (one week of pre-conditioning (week 0) and eight weeks of training (week 1 to week 8)). The pre-conditioning week was designed to allow volunteers to become familiar with all the exercises included in the training protocol. During the eight weeks of training, FG and CG carried out their workout session late in the afternoon or early evening. After a warm-up all subjects performed, in a randomized order, three circuits (Legs Circuit: leg extension, leg press, leg curl; Chest Circuit: pectoral machine, bench press, triceps machine; Back Circuit: vertical row, lat machine, biceps curl). Every exercise included 8 repetitions at 75% of 1RM each; four sets of each circuit were completed in about 1.5 h of training, with 4 min of recovery between the three circuits. All training sessions were closely monitored to ensure effort, repetitions and intensity established. All subjects complete all lifts for each exercise.

**Table 1:** Anthropometric and physical characteristics of the subjects at baseline. BMI, body mass index; 1RM, one repetition maximum. Values are presented as mean ± standard deviation. N.S., no significant differences between the groups, analyzed using a Fisher’s test (a) and an independent *t*-test (b).

	Food Group (*n* = 12)	Control Group (*n* = 14)	
Sex, M/F	8/4	11/3	N.S. ^(a)^
Age, years	23.7 ± 2.5	23.9 ± 4.2	N.S. ^(b)^
Weight, kg	67.8 ± 13.9	72.8 ± 14.2	N.S. ^(b)^
Height, cm	1.70 ± 0.06	1.73 ± 0.09	N.S. ^(b)^
BMI, kg/m^2^	23.5 ± 4.7	24.1 ± 3.5	N.S. ^(b)^
Fat Mass, kg	15.0 ± 6.7	15.9 ± 4.9	N.S. ^(b)^
Fat Free Mass, kg	52.8 ± 9.4	56.9 ± 11.9	N.S. ^(b)^
Lean Mass, kg	39.1 ± 7.3	41.1 ± 9.8	N.S. ^(b)^
1RM Bench Press, kg	51 ± 18	44 ± 18	N.S. ^(b)^
1RM Lat Machine, kg	58 ± 16	61 ± 18	N.S. ^(b)^
1RM Leg Press, kg	97 ± 24	93 ± 25	N.S. ^(b)^

### 2.4. Measures

#### 2.4.1. Strength Test

In order to test and measure chest, back and legs strength, respectively, a 1RM test was performed using BP, LAM and LP (Technogym SPA, Gambettola, Italy). The 1RM assessments were completed during pre-conditioning week, at the 4th week to adjust the loads of the training program and at the end of the study, for each participant, using a procedure prescribed by the American College of Sports Medicine [14]. Muscle strength was evaluated only on three main exercises (described above) because these involve all muscular groups used in the nine exercises of the training program.

#### 2.4.2. Body Composition Test

Body mass and stature were measured following standardized anthropometric protocols on a digital scale and an upright stadiometer (220 Seca, Deutschland) during the recruiting phase. Body mass index was calculated from these measures as kg/m^2^. FM, FFM and LM of all subjects were evaluated in the supine position, according to the National Institute of Health Consensus Statement [15], using a BIA (Model BIA 101, AKERN-RJL, Florence, Italy)*,* during pre-conditioning week and at the end of the study*.* There are highly statistically significant linear relationships between LM and FM assessed by BIA and more robust techniques (such as DEXA) in both sexes [16]. FFM was obtained by the subtraction of FM to total body mass and represent all “no fat” part of total body mass. LM is a part of FFM and its variation was considered as muscle mass variation. The parameters of body composition were calculated using manufacturer’s software BODY-GRAM PLUS 1.0^®^.

### 2.5. Statistical Methods

All data are reported as group means ± SD. At baseline the different males/female ratio between the FG and CG was analyzed using a Fisher’s test and the differences for anthropometric and physical characteristics between the groups was analyzed using an independent *t*-test (Table 1). All differences of the parameter assessed between week 0 and week 8 were calculated in both groups using a paired *t*-test (Table 2). Delta values of every parameter assessed between the groups were calculated using an independent *t*-test (Table 3). *p* values <0.05 were considered statistically significant.

## 3. Results

### 3.1. Body Composition

FM declined from week 0 to week 8 both in FG and in CG, but only in FG this decline was significant (week 0: 15.0 ± 6.7 kg; week 8: 13.1 ± 7.6 kg; Δ: −1.9 ± 2.9 kg; *p* < 0.05); FFM increased in all subjects, but only FG showed a significant increase (week 0: 52.8 kg ± 9.4; week 8: 55.1 kg ± 10.9; Δ: 2.3 ± 2.5 kg; *p* < 0.01). No significant differences in LM were found from week 0 to week 8 in either FG or CG, however there was an increase trend in FG and a decrease trend in CG (Table 2). No significant differences between groups in FM, FFM and LM were found at independent *t*-test on delta values (Table 3).

**Table 2:** Body composition in FG and CG: differences between week 0 and week 8 assessed by paired *t*-test; * *p* < 0.05 ** *p* < 0.01. FM, fat mass; FFM, fat free mass; LM, lean mass; FG, food group; CG, control group.

Group	*N*	FM (kg)		FFM (kg)		LM (kg)	
		Week 0	Week 8	Week 0	Week 8	Week 0	Week 8
FG	12	15.0 ± 6.7	13.1 ± 7.6 *	52.8 ± 9.4	55.1 ± 10.9 **	39.1 ± 7.3	40.3 ± 8.8
CG	14	15.9 ± 4.9	14.9 ± 5.6	56.9 ± 11.9	57.4 ± 11.4	41.1 ± 9.8	40.6 ± 9.3

**Table 3:** Differences between groups assessed by independent *t*-test on Δ values. FM, fat mass; FFM, fat free mass; LM, lean mass; BP, bench press; LAM, lat machine; LP, leg press; FG, food group; CG, control group.

Group	*N*	Δ FM	Δ FFM	Δ LM	Δ BP	Δ LAM	Δ LP
FG	12	−1.9 ± 2.9	2.3 ± 2.5	1.2 ± 2.4	11.8 ± 7.4	11.6 ± 6.2	76.6 ± 46.1
CG	14	−1.0 ± 2.2	0.5 ± 2.3	−0.5 ± 2.0	15.2 ± 11.5	9.0 ± 5.2	79.5 ± 36.4

### 3.2. Muscle Strength

**Figure 1:**
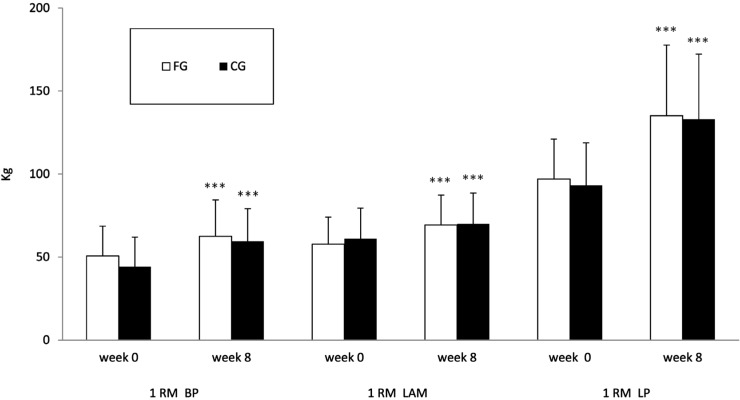
Muscle strength in FG and CG: differences between week 0 and week 8 assessed by paired *t*-test. *** *p* < 0.0001. FG, food group; CG, control group; 1RM BP, one repetition maximum on the bench press; 1RM LAM, one repetition maximum on the lat machine; 1RM LP, one repetition maximum on the leg press.

Muscular strength, as assessed by the 1RM bench press, lat machine and leg press, respectively, increased significantly for all subjects (23.3%, 20.1% and 39.5% in FG; 34.6%, 14.8% and 42.7% in CG) over the 8-week strength-training period (*p* < 0.0001). There were no significant differences between the FG and CG (Figure 1). No significant differences between the groups in 1RM were found at independent *t*-test on delta values (Table 3).

## 4. Discussion

The effect of lean beef coupled with resistance exercise on MPS is a new topic in sports nutrition. This was previously investigated in young, middle-aged and older subjects [10,11]. The studies available are very interesting, but as any other acute design study, they do not report possible changes in body composition and/or muscle strength. The aim of our experiment has been to evaluate whether consuming meat after exercise could result in an increase of LM during an eight weeks exercise program.

In our study we tried to understand if FM, FFM, LM and muscle strength could be affected during a resistance training program using meat protein instead of whey protein or commercial amino acids formulation.

In order to intake the typical post-workout protein serving size (20 g), we used 135 g of tinned beef with a low fat content (1.7 g), about 10 g of EAAs, 4.8 g of branched chain amino acids and more than 2 g of leucine. Leucine has been targeted as a key factor in translation initiation and the regulation of muscle protein synthesis [17]. The results showed in Table 3 indicate a significant decline of FM from week 0 to week 8 in FG group and an increase of FFM. The increase of FFM is a consequence of a change in FM, because no differences were found in LM during the trial. These effects are more likely to be due to an increase of protein intake rather than the temporal aspects of consumption. A link between daily protein intake and the loss of FM has been well established [18].

LM did not change in our study and this is probably due to two main reasons: (1) a low daily protein intake in the all subjects. As we know, in order to produce muscle hypertrophy, the daily effective diet should contain ~1.8–2.0 g of protein per kilogram of body mass [5]; (2) the peak plasma concentrations of EAAs after 135 g of beef ingestion occurred more slowly (approximately equaling to 120 min), as recently reported [19], compared to that of more rapidly digested and absorbed free amino acids or whey protein supplements. This certainly reduces the rapid availability of EAAs soon after the exercise session, leading to a partial miss of the anabolic window and therefore the “temporal” effect of the meat supplementation on the development of muscle mass. Taken together, these considerations lead us to speculate that an increase of LM through a long-term beef supplementation strategy may only be possible by increasing of a total daily protein intake (at least 1.8 g/kg/day with an energy balanced diet) and consuming the meat before exercise. As the authors discussed, in terms of plasma amino acid precursor supply and ultimately protein synthesis response, consuming a slowly digested protein-rich mixed meal 60 min prior to exercise, may be the physiological equivalent of ingesting a rapidly digested protein source (e.g., whey protein) 30–60 min post-exercise [10].

Muscular strength, as assessed by the 1RM bench press, lat machine and leg press, respectively, increased significantly in both groups (Figure 1); this effect was independent of LM values and nutritional stimulus, but only related to the resistance-training program.

Despite the present study including both men and women to maximize the potential generalizability of the findings and the different composition of the groups (males/female ratio) has not shown statistical significance in the results, gender is an important consideration in interpreting responses to interventions for body composition changing and muscle adaptations. Future studies are therefore needed to elucidate these aspects, trying to overcome some limitations of this research, such as the sample size and study duration.

## 5. Conclusions

In conclusion, the consumption of beef protein from tinned meat could be considered a nutrition strategy in addition to other proteins or amino acid supplements, useful for guaranteeing high essential amino acids availability close to exercise. Tinned meat is more digestible than other meat sources (e.g., steak), does not generally cause any gastrointestinal distress and its consumption is also practical. However, according to previous research [10,19] and based on this study, meat supplementation should probably be done at least 1 h before a resistance exercise session. The use of meat as a supplement should not exceed two or three times per week, according to general dietary guidelines that recommend a moderate consumption of red meat.

## Key Acronyms

MPS: Muscle protein synthesis
EAAs: essential amino acids
1RM: one repetition maximum
FG: food group
GP: control group
FM: fat mass
FFM: fat free mass
LM: lean mass
BIA: bioelectrical impedance analyzer
BP: bench press
LAM: lat machine
LP: leg press
BMI: body mass index
